# Accuracy of the COPD diagnostic questionnaire as a screening tool in primary care

**DOI:** 10.1186/s12875-022-01685-z

**Published:** 2022-04-14

**Authors:** Lisa Pagano, Zoe McKeough, Sally Wootton, Nicholas Zwar, Sarah Dennis

**Affiliations:** 1 Sydney School of Health Sciences, Faculty of Medicine and Health, University of Sydney, Camperdown, Sydney, NSW 2006 Australia; 2 Chronic Disease Community Rehabilitation Service, Northern Sydney Local Health District, Sydney, Australia; 3 Faculty of Health Sciences and Medicine, Bond University, Gold Coast, Australia; 4 Ingham Institute for Applied Medical Research, Liverpool, Australia; 5 South Western Sydney Local Health District, Liverpool, Australia

## Abstract

**Background:**

The COPD Diagnostic Questionnaire (CDQ) was developed to identify people who would benefit from spirometry testing to confirm Chronic Obstructive Pulmonary Disease (COPD). The aim of this study was to determine the usefulness of a cut-off score of 16.5 on the CDQ in identifying those at increased risk of obstruction, in a mixed population of people ‘at risk’ of COPD and those with an ‘existing’ COPD diagnosis.

**Methods:**

People ‘at risk’ of COPD (aged > 40 years, current/ex-smoker) and those with ‘existing’ COPD were identified from four general practices and invited to participate. Participants completed the CDQ and those with a CDQ score ≥ 16.5 were categorised as having intermediate to increased likelihood of airflow obstruction. Pre and post-bronchodilator spirometry determined the presence of airway obstruction (FEV_1_/FVC ratio < 0.7). Sensitivity, specificity and accuracy of the CDQ was determined compared to spirometry as the gold standard.

**Results:**

One hundred forty-one participants attended an initial assessment (‘at risk’ = 111 (79%), ‘existing’ COPD = 30 (21%)). A cut-off score of 16.5 corresponded to a sensitivity of 81%, specificity of 36% and accuracy of 50%, in the entire mixed population. The area under the ROC curve was 0.59 ± 0.50 indicating low diagnostic accuracy of the CDQ. Similar results were found in the ‘existing’ COPD group alone.

**Conclusion:**

Whilst a cut-off score of 16.5 on the CDQ may result in a large number of false positives, clinicians may still wish to use the CDQ to refine who receives spirometry due to its high sensitivity.

**Trial registration:**

ANZCTR, ACTRN12619001127190. Registered 12 August 2019 – Retrospectively registered, http://www.ANZCTR.org.au/ACTRN12619001127190.aspx

## Background

Chronic obstructive pulmonary disease (COPD) places a significant burden on healthcare systems worldwide and is a leading cause of mortality and health care expenditure [1, 2]. COPD is also a leading cause of potentially preventable hospitalisations in Australia [3] in which admissions could be prevented by timely and optimal health care in the community [4]. Preventative health interventions, such as smoking cessation and promotion of physical activity, have been shown to have positive outcomes for people with COPD, in terms of reducing the risk of all-cause mortality and hospital admissions, as well as improving health-related quality of life [5–9]. If COPD can be detected early, then implementation of these preventative health strategies could help to reduce the burden on the healthcare system by keeping people healthy for longer, thereby preventing exacerbations and hospitalisations.

The Global Initiative for Chronic Obstructive Lung Disease (GOLD) guidelines advocate for early detection of COPD [2]. Many people with chronic or persistent respiratory symptoms present to a general practitioner (GP) before a respiratory physician, which places primary care in an ideal position for the early diagnosis of COPD [10]. Despite this, there is evidence that COPD is currently underdiagnosed in primary care [11–14]. Poor utilisation of spirometry in general practice could contribute to this, with some studies reporting that up to 50% of patients with respiratory symptoms do not undergo spirometry testing [15–17]. Reported challenges to performance of spirometry include lack of time, lack of access to resources such as working spirometers and also training in performance of spirometry [18–20]. An additional challenge for the GP is that patients can often adapt to the insidious onset of COPD symptoms and may only seek medical advice when symptoms impair their quality of life, potentially delaying diagnosis and optimisation of treatment [21–23].

Several studies have also demonstrated high rates of misdiagnosis of COPD in general practice, with a high proportion of patients with an existing COPD diagnosis not meeting the spirometric definition of COPD [13, 19, 24–26]. This could relate to diagnosis often being based on clinical symptoms rather than gold standard spirometry testing or problems with quality or interpretation of spirometry in primary care [18, 26–28]. For example, one Australian study completed in 31 general practices found that spirometric confirmation was not present in 31% of cases of COPD [29]. Strategies are needed in primary care to improve the diagnosis of COPD, including not only improved detection, but also the accuracy and confirmation of an existing diagnosis.

Active case finding of ‘at risk’ patients is one way to improve the early diagnosis of COPD [2]. Widespread spirometric testing could be useful as pre and post-bronchodilator spirometry with a post-bronchodilator forced expiratory volume in 1 second (FEV_1_)/forced vital capacity (FVC) of < 0.7 remains the gold standard for a diagnosis of COPD [2, 10]. However, pre-selection of patients who present with symptoms or have risk factors may result in more economical utilisation of healthcare resources, by reducing unwarranted spirometry testing. Microspirometry has been shown to be a sensitive and effective tool to use in primary care to detect cases of undiagnosed airflow obstruction [30, 31]. Yet the use of microspirometry in primary care may present with similar barriers to spirometry including access to equipment and training in the use of the device.

Symptom-based questionnaires, which identify factors such as smoking history and symptoms commonly associated with COPD, may be helpful as an initial screening tool to identify those patients who have an increased likelihood of having airflow obstruction and would likely benefit from spirometry testing [32]. The COPD Diagnostic Questionnaire (CDQ) is one such screening tool developed for use in primary care [33, 34]. This 8-item tool comprises questions regarding demographics, smoking history and symptoms, and was designed for use in patients aged 40 years and over with a history of smoking, but no prior respiratory diagnosis or differential diagnosis. Participants receive a score out of 38 and subjects are then classified as being at decreased likelihood (0 < 16.5 points), intermediate likelihood (≥16.5–< 19.5 points) or increased likelihood (≥19.5 points) of airflow obstruction. Price and colleagues (2006) proposed that those at increased likelihood of obstruction should undergo spirometry, and those at decreased likelihood would not require spirometry in most cases, with a reasonable degree of certainty [34].

In the initial development of the CDQ by Price et al. (2006), accuracy was reported to be high according to receiver operating characteristic (ROC) analysis, with an area under the curve (AUC) of 0.82 [33]. In a following paper examining the scoring system and clinical application of the CDQ, this group reported a sensitivity of 58.7% and specificity of 77.0% when applying a cut-off score of 16.5 [34]. Since then, external validation studies of the CDQ have reported mixed results on the accuracy of the CDQ at detecting patients at risk of airflow obstruction. Reported sensitivities have ranged from 79.7 to 93.9% for 16.5 as a cut-off score with corresponding low specificities, in a population of people at risk of airflow obstruction [35–39]. There are currently no studies examining the accuracy of the CDQ in detecting the presence of airflow obstruction in people with an existing COPD diagnosis, to assist clinicians in confirming a diagnosis of COPD. Therefore, the primary aim of this study was to determine the sensitivity, specificity and accuracy of using a cut-off score of 16.5 on the CDQ as an indicator of airflow obstruction, in a mixed population of people ‘at risk’ of COPD or with ‘existing’ COPD. Secondary aims were to a) determine the sensitivity, specificity and accuracy of a cut-off score of 16.5 on the CDQ as an indicator of airflow obstruction in the ‘existing’ COPD group alone, and b) determine the optimal cut-off score using ROC curve analysis in a population of people ‘at risk’ of COPD and ‘existing’ COPD.

## Methods

This cross-sectional study was embedded in a larger pilot study evaluating a GP-Physiotherapist partnership for COPD in primary care. The detailed methods have been published previously [40]. In brief, four general practices were selected from a metropolitan Sydney Primary Health Network and consented to participate. Potentially eligible participants at risk of COPD or with an existing diagnosis of COPD were identified from a search of the general practice databases and invited to take part in the study via telephone or letter. Two groups of participants were included: those ‘at risk’ of COPD and those with ‘existing’ COPD. Participants were considered eligible if they: (i) were adults aged 40 years and over; (ii) had attended the practice at least twice with one visit in the preceding 12 months; and (iii) had a documented history of smoking (current or former smoker) in their medical notes or (iv) had a recorded diagnosis of COPD or were taking medications prescribed for COPD (i.e. short acting inhaled β_2_ agonists (SABA), short acting muscarinic antagonists (SAMA), long acting inhaled β_2_ agonists (LABA), long acting muscarinic antagonists (LAMA), combination of LABA/LAMA and inhaled corticosteroids). The list of potential participants was screened by GPs or practice nurses and participants were excluded if they had terminal cancer, a cognitive impairment, required home oxygen, were unable to understand sufficient English or were pregnant.

After obtaining written informed consent, participants were invited to attend an assessment with a senior respiratory physiotherapist at the general practice. At the assessment, participants completed the CDQ and were classified as having decreased likelihood (0 < 16.5 points), intermediate likelihood (≥16.5–< 19.5 points) or increased likelihood (≥19.5 points) of airflow obstruction. Pre and post-bronchodilator spirometry according to the American Thoracic Society/European Respiratory Society (ATS/ERS) lung function guidelines [41] was completed by the physiotherapist. A diagnosis of COPD based on GOLD guidelines was assigned to all those participants with a post-bronchodilator FEV_1_/FVC of < 0.7 [2].

The study protocol was approved by the Northern Sydney Local Health District Human Research Ethics committee (HREC reference; HREC/15/HAWKE/434) and was conducted in accordance with the WMA Declaration of Helsinki. The study protocol was registered at the Australia New Zealand Clinical Trials Registry (ACTRN12619001127190).

### Statistical analysis

The sum score for the CDQ was calculated based on the original scoring protocol outlined by Price et al. (2006) [34]. A 2 × 2 contingency table was used for the entire mixed population to calculate the sensitivity, specificity, accuracy, positive likelihood ratio (+LR) and negative likelihood ratio (−LR) for the cut-off score of 16.5 on the CDQ at detecting those at intermediate to increased risk of COPD. A second 2 × 2 contingency table was used for the ‘existing’ COPD cohort alone to also calculate the sensitivity, specificity and accuracy of a score of 16.5 on the CDQ at detecting airflow obstruction. The participants were classified according to their “predicted” likelihood of obstruction from the CDQ results and their “actual” obstruction from the post-bronchodilator spirometry results.

The discriminatory ability and accuracy of the CDQ was determined using ROC curve analysis. For calculation of the ROC curve, the raw CDQ scores were used as the screening test variable and a diagnosis of COPD based on post-bronchodilator spirometry results, as the classification variable. The AUC value was analysed to determine the discriminative ability of the CDQ across the full range of cut-off scores: high accuracy (AUC > 0.9), moderate accuracy (AUC 0.7 to 0.9), low accuracy (AUC 0.5 to 0.7) or a chance result (AUC < 0.5) [42, 43]. The sensitivity and specificity of different cut-points were also analysed using ROC curve data to determine the score with optimal balance of sensitivity and specificity. Data was analysed using IBM SPSS Statistics for Windows, version 24.0. (IBM Corp., Armonk, N.Y., USA).

## Results

**Fig. 1:**
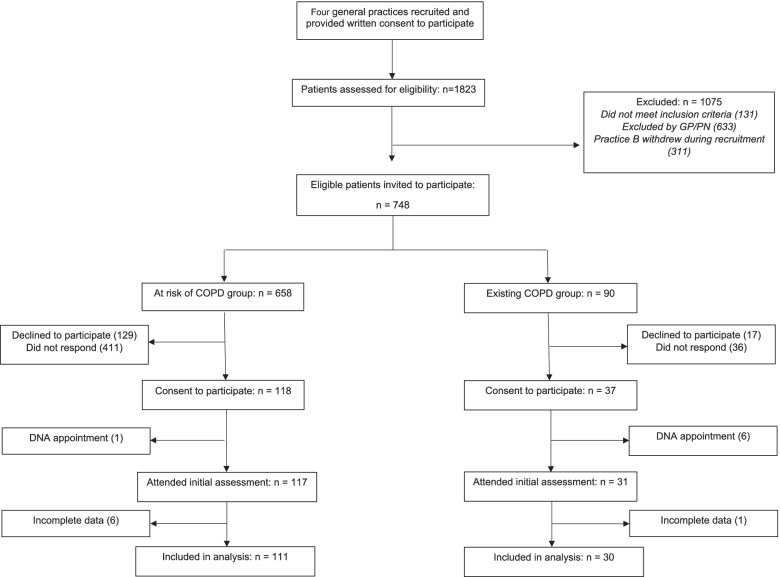
Study Enrolment. Abbreviations: COPD: Chronic Obstructive Pulmonary Disease; DNA: did not attend

Data on participant flow is shown in Fig. 1. A total of 748 eligible participants were invited to participate. Of these, 658 were ‘at risk’ of COPD and 90 had an ‘existing’ COPD diagnosis. Of those invited to participate, 146 declined to participate and 447 did not respond. A total of 148 participants provided written informed consent and attended a baseline assessment with the physiotherapist. Of these, 141 (95%) participants had complete CDQ data recorded and spirometry meeting criteria for analysis [41] and so these participants were included in the data analysis.

Demographic characteristics of the participants is presented in Table 1. There were 111 participants classified as ‘at risk’ of COPD and 30 participants had an ‘existing’ COPD diagnosis. Based on post-bronchodilator spirometry results, 30% (*n* = 42) of participants demonstrated airflow obstruction and were diagnosed as having COPD (*n* = 18 in the ‘at risk’ group, *n* = 24 in the ‘existing’ COPD group). According to GOLD criteria for classification of COPD severity [2], the majority of participants with airflow obstruction were classified as GOLD Stage II (*n* = 26). The mean CDQ score for the total cohort was 19.1 (SD 5.6).

**Table 1:** Population characteristics of subjects

	AT RISK of COPD ***n*** = 111	EXISTING COPD ***n*** = 30	TOTAL ***n*** = 141
Age (years; mean ± SD)	67 (11.4)	71 (10.3)	68 (11.2)
Gender (% female)	57 (51%)	24 (80%)	81 (57%)
Body mass index (Kg/m^2^; mean ± SD)	27.8 (5.2)	28.2 (5.6)	27.9 (5.3)
Current smokers	10 (9%)	6 (20%)	16 (11%)
Former smokers	101 (91%)	18 (60%)	119 (84%)
Never smoked	0 (0%)	6 (20%)	6 (4%)
CDQ score (mean ± SD)	18.9 (5.6)	20.0 (5.5)	19.1 (5.6)
CDQ score ≥ 16.5	73 (66%)	24 (80%)	97 (69%)
CDQ score ≥ 19.5	43 (39%)	16 (53%)	59 (42%)
Post-bronchodilator FEV_1_/FVC (mean ± SD)	0.78 (0.8)	0.62 (0.1)	0.74 (0.1)
Post-bronchodilator FEV_1_ (mean ± SD)	2.67 (0.8)	1.63 (0.7)	2.45 (0.9)
Airway obstruction (FEV_1_/FVC ≤ 0.7)	18 (16%)	24 (80%)	42 (30%)
Frequency (%)			
Current smokers	3 (3%)	6 (20%)	9 (6%)
Former smokers	15 (14%)	14 (47%)	29 (21%)
Never smoked	0 (0%)	4 (13%)	4 (3%)
GOLD Stage I	9 (8%)	4 (13%)	13 (9%)
GOLD Stage II	9 (8%)	17 (57%)	26 (18%)
GOLD Stage III	0 (0%)	2 (7%)	2 (1%)
GOLD Stage IV	0 (0%)	0 (0%)	0 (0%)
No obstruction Frequency (%)	93 (84%)	6 (20%)	99 (70%)
Current smokers	7 (6%)	0 (0%)	7 (5%)
Former smokers	86 (77%)	4 (13%)	90 (64%)
Never smoked	0 (0%)	2 (7%)	2 (1%)

Data are presented as Number (%) unless indicated otherwise

*Abbreviations*: *CDQ* COPD Diagnostic Questionnaire, *COPD* Chronic Obstructive Pulmonary Disease, *FEV*_1_ Forced expiratory volume in one second, *FVC* Forced vital capacity, *GOLD* Global Initiative for Chronic Obstructive Lung Disease

COPD GOLD staging classification^2^ - Stage 1: FEV_1_ ≥ 80%; Stage 2: FEV_1_ 50-79%; Stage 3: FEV_1_ 30-49%; Stage 4: FEV_1_ < 30%

**Fig. 2:**
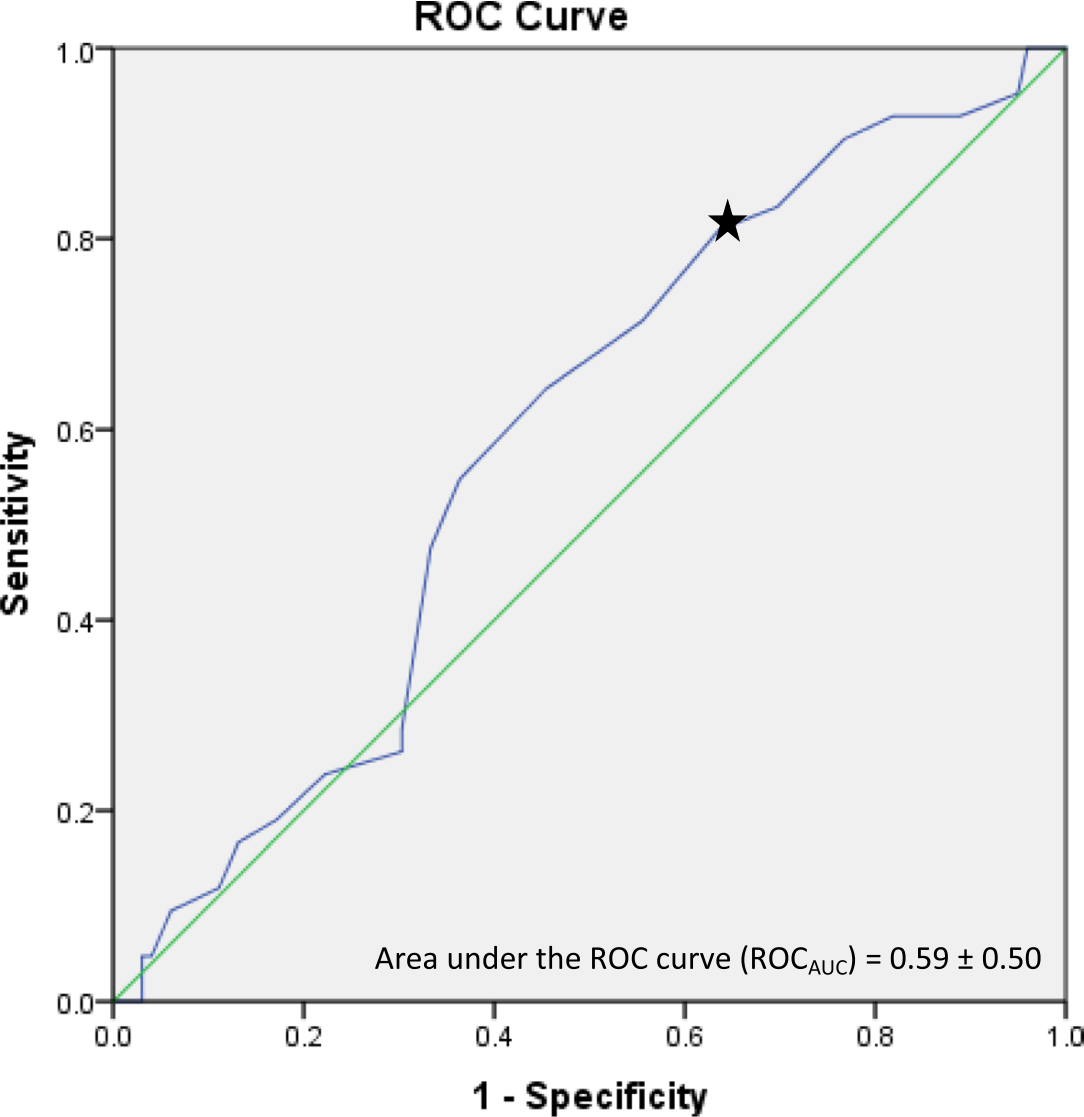
Receiver operating characteristic (ROC) curve comparing CDQ score to COPD diagnosis using GOLD spirometry criteria for obstruction (post-bronchodilator FEV1/FVC < 0.70) as the reference standard. Area under the ROC curve (ROCAUC) = 0.59 ± 0.50. A ROCAUC of 0.5 is indicated by the solid diagonal line. Cut point 16.5 is indicated by the black star>

Sensitivity and specificity analyses for the CDQ cut-off score of 16.5 for the entire mixed population (‘at risk’ of COPD and ‘existing’ COPD) is presented in Table 2. Ninety-seven (69%) participants scored ≥16.5 and were predicted to have an intermediate to increased likelihood of airflow obstruction. The remaining 44 (31%) participants scored < 16.5 and were predicted to have a reduced likelihood of obstruction on spirometry. Sensitivity and specificity for the 16.5 cut-off point were 81.0 and 36.4% respectively, whilst accuracy of this cut-off score was determined to be 50.0%. The +LR was 1.27, indicating that a score of ≥16.5 on the CDQ is 1.27 times more likely among patients with obstruction than those without obstruction. The -LR was 0.52, indicating that someone with airway obstruction is about half as likely to score ≥ 16.5 on the CDQ than someone without obstruction. The ROC_AUC_ was 0.59 ± 0.50 (shown in Fig. 2), indicating overall poor diagnostic accuracy of the CDQ as a screening tool.

**Table 2:** 2 × 2 contingency table detailing the sensitivity and specificity analyses for the CDQ cut off score of 16.5 for subjects in ‘at risk’ and ‘existing’ COPD groups

	True class (COPD diagnosis with presence of obstruction confirmed by spirometry)		
	True	False	Total
Predicted class (Score ≥ 16.5)			
True	34 (24.1%)	63 (44.7%)	97 (68.8%)
False	8 (5.7%)	36 (25.5%)	44 (31.2%)
Total	42 (29.8%)	99 (70.2%)	141 (100.0%)

Sensitivity and specificity analyses for the CDQ cut off score of 16.5 in our cohort of ‘existing’ COPD participants is presented in Table 3. Sensitivity for the 16.5 cut-off point in this cohort was determined to be 83.3%, whilst specificity was 33.3% and accuracy was 73.3%.

**Table 3:** 2 × 2 contingency table detailing the sensitivity and specificity analyses for the CDQ cut off score of 16.5 for ‘existing’ cases of COPD

	True class (COPD diagnosis with presence of obstruction confirmed by spirometry)		
	True	False	Total
Predicted class (Score ≥ 16.5)			
True	20 (66.7%)	4 (13.3%)	24 (80.0%)
False	4 (13.3%)	2 (6.7%)	6 (20.0%)
Total	24 (80.0%)	6 (20.0%)	30 (100.0%)

## Discussion

In this paper we examined the usefulness of the CDQ as a screening tool in primary care to identify within a mixed population of patients, those at increased likelihood of COPD. When applying the cut-off point of 16.5 to categorise patients in terms of their likelihood of obstruction [34], the sensitivity was 81.0%, indicating that this score could be used to identify whom would benefit from spirometry. However, the corresponding specificity was low at only 36.4%, with accuracy at 50.0%. This means that in our mixed cohort, whilst the CDQ was sensitive and identified those who truly had obstruction, there were a high number of false positives with approximately 45% of the population having undergone testing, with no evidence of obstruction on spirometry. In addition, accuracy of the CDQ for people with previous doctor diagnosed COPD was only 73%, highlighting potential issues with detecting airflow obstruction, as well as misdiagnosis.

In the initial development of the CDQ in COPD, Price and colleagues (2006) reported overall high accuracy of the CDQ (ROC_AUC_ 0.82) [33] and when applying a cut-off score of 16.5, moderate sensitivity and high specificity [34]. In contrast, in our mixed population of ‘at risk’ of COPD and ‘existing’ COPD when using the same cut-off point, we found high sensitivity and low specificity, as well as poor overall diagnostic accuracy (ROC_AUC_ 0.59 ± 0.50). The resulting likelihood ratios further indicate limited utility of this cut-off score at detecting those at risk of obstruction. According to our results, when utilising the +LR, the pre-test probability of obstruction of 30% would be associated with a very small increase in post-test probability to 35%. Similarly, when looking at the –LR, the same pre-test probability of 30%, would be associated with a small reduction in post-test probability to 18%. This reflects the low specificity and high rate of false positives when using 16.5 as a cut-off score in our mixed cohort.

Other studies examining the CDQ have also reported similar findings of moderate to high sensitivity (79.7 to 93.9%) but low specificity (24.4 to 46.8%) and accuracy (ROC_AUC_ 0.65 to 0.79) of the cut-off score of 16.5 [35–39]. The reasons for these differences in results from the original sample [33] used to develop and validate the CDQ are not readily apparent. Discrepancies could be due to different populations utilised by each study. When examining our cohort, there were some differences compared to the original sample [33]. Our cohort was older (68 ± 11 vs 58 ± 11 years), with lower cigarette consumption (21 ± 19 vs 26 ± 24 pack years) and comprised a lower number of current smokers compared to former smokers. Considering that higher scores are allocated to higher pack years and higher age, this may have impacted results. For example, approximately 45% of our population reported a pack-year history of 14 years or less of which only 25% had airflow obstruction on spirometry. This is in comparison to 34% of confirmed cases that reported a 25 to 49 pack-year history. Although our study population was also mixed (i.e. ‘at risk’ of COPD and ‘existing’ COPD), when comparing baseline characteristics of the two groups (Table 1), the groups were comparable except for worse lung function in the ‘existing’ COPD group (FEV_1_/FVC 0.62 vs 0.78). If the CDQ was truly accurate at discriminating between those with and without obstruction, it would be feasible to postulate that our sample should have produced results with higher overall accuracy similar to the original sample used for the development and validation of the CDQ [33, 34], given that our sample contained participants with known obstruction.

A secondary aim of this study was to review the sensitivity, specificity and accuracy of a cut-score of 16.5 in the ‘existing’ COPD cohort alone. In this population, the results were similar to our mixed cohort with a sensitivity and specificity of 83.3 and 33.3% respectively. Whilst accuracy was higher compared to the ‘mixed’ group, four existing cases of COPD were not detected. One would have anticipated that the ‘existing’ COPD population would have produced higher CDQ scores over the 16.5 cut-off, however, only 80% of this cohort had a score ≥ 16.5. Furthermore, six participants with a previous diagnosis of COPD did not show obstruction on spirometry. This is not surprising as there is evidence of high rates of misdiagnosis of COPD in primary care [13, 19, 24–26]. Yet, the CDQ was only able to correctly identify two of these cases as true negatives, whilst the remaining four cases would still have undergone spirometry testing. Despite the small sample size of this group, these results add further weight to the concerns we have with the CDQ as a screening tool in identifying those who may have obstruction.

There is debate in the literature about whether a higher or lower cut-off score on the CDQ is more useful in screening for COPD [36, 37, 44]. Literature suggests that a diagnostic test that is most useful at discriminating between people with and without a disease should combine high levels of sensitivity and specificity [45, 46]. The original study [34] proposed that those who score less than 16.5 on the CDQ are less likely to have obstruction, and those who score ≥ 19.5 are at high risk of obstruction and should undergo spirometry. Of those that fall in the intermediate zone of scoring ≥16.5 to < 19.5, the decision to undergo spirometry is left to the clinician. This decision is due to the different healthcare contexts clinicians may be faced with in terms of budget, resources and policy. Our ROC curve, as seen in Fig. 2, is fairly linear indicating there is some evidence that multiple scores between 13.5 and 19.5 could be considered for use as a cut-off score. Yet the implications of using a higher or lower cut-off score must be considered. A lower cut-off score will have higher sensitivity yet, would also require a substantial increase in the number of spirometry tests being performed and subsequent false positives. For example, according to our ROC curve, a score of 15.5 when compared to a score of 16.5, would lead to a decrease in specificity by approximately 5%, with only a 2% gain in sensitivity. This means that seven additional spirometry tests would have to be performed in order to detect one additional case of COPD. Alternatively, whilst a higher cut-off score may lower the number of false positives, COPD cases may be missed.

The choice of optimal cut-off score on the CDQ may come down to clinician preference and patient presentation. For example, clinicians who are concerned with missing potential diagnoses and those that have the resources and time available may prefer to use a lower cut-off score. Yet, literature suggests that the use of spirometry in primary care is low [15–17] with GPs citing time constraints and availability of resources as barriers to completing spirometry [18, 47, 48]. As such, GPs may potentially be more inclined to use a higher cut-off score of 19.5 for screening to ensure that COPD is more likely to be identified. However, when you examine our ROC curve, the sensitivity and specificity when using 19.5 as the cut-off score was 54.8 and 63.6% respectively and this is similar to other studies [35–39]. Whilst this would result in less spirometries required, sensitivity would decrease by 26%, such that 19 cases of COPD would have been missed compared with only 8 cases when 16.5 is used as the cut-off score. Furthermore, it is important to consider that a screening tool that utilises a lower cut-off point with higher sensitivity may be more desirable when the consequence of a false negative exceeds the consequence of a false positive. For example, screening more people at risk of COPD may still be useful as it can provide the clinician with the opportunity to provide preventative lifestyle interventions, which are likely to benefit the patient if adopted, such as physical activity and smoking cessation advice.

An additional consideration is the need to address the underdiagnosis and misdiagnosis of COPD in primary care, by providing strategies for clinicians to screen for symptoms and refer those at increased risk of fixed obstruction for spirometry testing. The use of handheld flow meters under the supervision of trained health professionals was found to be significantly more accurate than the CDQ for discriminating between smokers with and without airway obstruction [30, 38, 39]. Yet, due to the high sensitivity of the CDQ as well as the practical advantages of being low cost, readily available and easy to use, the CDQ could still assist clinicians to target the use of spirometry on patients at greater risk of COPD to improve the efficiency and accuracy of COPD diagnosis. A patient can complete the CDQ with minimal assistance prior to attending an appointment at low cost versus a handheld flow meter which would require a trained clinician to be present.

There were some limitations to this study. Firstly, only a small number of general practices were involved with a small sample size of patients which may limit the generalisability of findings to the broader COPD population. Recruitment of patients was ceased due to COVID-19 in January 2020 which also affected sample size. Secondly, identifying potentially eligible patients from practice records based on smoking status may have affected recruitment as smoking status is not always accurately recorded.

## Conclusion

The results of this study suggest that a score of 16.5 on the CDQ was sensitive to identify those who would benefit from diagnostic spirometry. Therefore, this cut-off score could be used by clinicians to screen for those at high risk of obstruction, without missing too many cases of COPD. However, clinicians should be aware that there will be a high number of false positives due to the low specificity of this cut-off score. In addition, whilst our results suggest overall low diagnostic accuracy of the CDQ, the CDQ may have a place in clinical practice to assist clinicians in determining who would be most appropriate for spirometry and negating the need to perform spirometry on all patients but may not have a place in confirming an existing COPD diagnosis.

## Data Availability

Data will be stored according as required by the ethics committee and will be available from the authors on request.

## Abbreviations

ATS: American Thoracic Society
AUC: Area under the curve
CDQ: COPD Diagnostic Questionnaire
COPD: Chronic Obstructive Pulmonary Disease
ERS: European Respiratory Society
FEV1: Forced expiratory volume in one second
FVC: Forced vital capacity
GOLD: Global Initiative for Chronic Obstructive Lung Disease
GPs: General Practitioners
LABA: Long-acting beta2-agonists
LAMA: Long acting muscarinic antagonists
ROC: Receiver operating characteristic
SABA: Short-acting beta2-agonists
SAMA: Short-acting muscarinic antagonists
WMA: World Medical Association
+LR: Positive likelihood ratio
-LR: Negative likelihood ratio

## References

[1] World Health Organization. Global health estimates: leading causes of death. Geneva: WHO (World Health Organisation); 2020. [Available from: https://www.who.int/data/gho/data/themes/mortality-and-global-health-estimates/ghe-leading-causes-of-death (Accessed 12 Jan 2021).

[2] Global Initiative for Chronic Obstructive Lung Disease. Global strategy for the diagnosis, management and prevention of chronic obstructive pulmonary disease: 2021 report (2020). Retrieved from: https://goldcopdorg/wp-content/uploads/2020/11/GOLD-REPORT-2021-v11-25Nov20-WMV.pdf (Accessed 12 Jan 2021).

[3] Australian Institute of Health and Welfare (2019). Potentially preventable hospitalisations in Australia by age groups and small geographic areas, 2017–18.

[4] Australian Institute of Health and Welfare (2020). Disparities in potentially preventable hospitilisations across Australia, 2012-13 to 2017-18.

[5] McKeough Z, Cheng SWM, Alison J, Jenkins C, Hamer M, Stamatakis E (2018). Low leisure-based sitting time and being physically active were associated with reduced odds of death and diabetes in people with chronic obstructive pulmonary disease: a cohort study. J Phys.

[6] Garcia-Aymerich J, Lange P, Benet M, Schnohr P, Antó JM (2006). Regular physical activity reduces hospital admission and mortality in chronic obstructive pulmonary disease: a population based cohort study. Thorax.

[7] Godtfredsen N, Vestbo J, Osler M, Prescott E (2002). Risk of hospital admission for COPD following smoking cessation and reduction: a Danish population study. Thorax.

[8] Godtfredsen NS, Lam TH, Hansel TT, Leon M, Gray N, Dresler C (2008). COPD-related morbidity and mortality after smoking cessation: status of the evidence. Eur Respir J.

[9] McCarthy B, Casey D, Devane D, Murphy K, Murphy E, Lacasse Y (2015). Pulmonary rehabilitation for chronic obstructive pulmonary disease. Cochrane Database Syst Rev.

[10] Yang IABJ, George J, Jenkins S, McDonald CF, McDonald V, Smith B, Zwar N, Dabscheck E (2020). The COPD-X plan: Australian and New Zealand guidelines for the management of chronic obstructive pulmonary disease.

[11] Toelle BG, Xuan W, Bird TE, Abramson MJ, Atkinson DN, Burton DL (2013). Respiratory symptoms and illness in older Australians: the burden of obstructive lung disease (BOLD) study. Med J Aust.

[12] Bednarek M, Maciejewski J, Wozniak M, Kuca P, Zielinski J (2008). Prevalence, severity and underdiagnosis of COPD in the primary care setting. Thorax.

[13] Hill K, Goldstein RS, Guyatt GH, Blouin M, Tan WC, Davis LL (2010). Prevalence and underdiagnosis of chronic obstructive pulmonary disease among patients at risk in primary care. Cmaj.

[14] Lamprecht B, Soriano JB, Studnicka M, Kaiser B, Vanfleteren LE, Gnatiuc L (2015). Determinants of underdiagnosis of COPD in national and international surveys. Chest.

[15] Joo MJ, Au DH, Fitzgibbon ML, McKell J, Lee TA (2011). Determinants of spirometry use and accuracy of COPD diagnosis in primary care. J Gen Intern Med.

[16] Miravitlles M, de la Roza C, Naberan K, Lamban M, Gobartt E, Martin A (2007). Use of spirometry and patterns of prescribing in COPD in primary care. Respir Med.

[17] Walters JA, Hansen EC, Johns DP, Blizzard EL, Walters EH, Wood-Baker R (2008). A mixed methods study to compare models of spirometry delivery in primary care for patients at risk of COPD. Thorax.

[18] Joo MJ, Sharp LK, Au DH, Lee TA, Fitzgibbon ML (2013). Use of spirometry in the diagnosis of COPD: a qualitative study in primary care. COPD: J Chron Obstruct Pulmon Dis.

[19] Zwar NA, Marks GB, Hermiz O, Middleton S, Comino EJ, Hasan I (2011). Predictors of accuracy of diagnosis of chronic obstructive pulmonary disease in general practice. Med J Aust.

[20] Salinas GD, Williamson JC, Kalhan R, Thomashow B, Scheckermann JL, Walsh J (2011). Barriers to adherence to chronic obstructive pulmonary disease guidelines by primary care physicians. Int J Chron Obstruct Pulmon Dis.

[21] Fromer L (2011). Diagnosing and treating COPD: understanding the challenges and finding solutions. Int J Gen Med.

[22] Tinkelman DG, Price DB, Nordyke RJ, Halbert RJ (2007). COPD screening efforts in primary care: what is the yield?. Prim Care Respir J.

[23] Van Schayck C, Van der Heijden F, van Den Boom G, Tirimanna P, Van Herwaarden C (2000). Underdiagnosis of asthma: is the doctor or the patient to blame? The DIMCA project. Thorax.

[24] Sichletidis L, Chloros D, Spyratos D, Chatzidimitriou N, Chatziiliadis P, Protopappas N (2007). The validity of the diagnosis of chronic obstructive pulmonary disease in general practice. Prim Care Respir J.

[25] Liang J, Abramson MJ, Zwar NA, Russell GM, Holland AE, Bonevski B (2018). Diagnosing COPD and supporting smoking cessation in general practice: evidence-practice gaps. Med J Aust.

[26] Zwar NA, Bunker JM, Reddel HK, Dennis SM, Middleton S, van Schayck OC (2016). Early intervention for chronic obstructive pulmonary disease by practice nurse and GP teams: a cluster randomized trial. Fam Pract.

[27] Walters JA, Hansen E, Mudge P, Johns DP, Walters EH, Wood-Baker R (2005). Barriers to the use of spirometry in general practice. Aust Fam Physician.

[28] Fisk M, McMillan V, Brown J, Holzhauer-Barrie J, Khan MS, Baxter N (2019). Inaccurate diagnosis of COPD: the welsh national COPD audit. Br J Gen Pract.

[29] Walters JA, Walters EH, Nelson M, Robinson A, Scott J, Turner P (2011). Factors associated with misdiagnosis of COPD in primary care. Prim Care Respir J.

[30] Schnieders E, Ünal E, Winkler V, Dambach P, Louis VR, Horstick O (2021). Performance of alternative COPD case-finding tools: a systematic review and meta-analysis. Eur Respir Rev.

[31] Schermer TR, Vatsolaki M, Behr R, Grootens J, Cretier R, Akkermans R (2018). Point of care microspirometry to facilitate the COPD diagnostic process in primary care: a clustered randomised trial. NPJ Prim Care Respir Med.

[32] Van Schayck C, Loozen J, Wagena E, Akkermans R, Wesseling G (2002). Detecting patients at a high risk of developing chronic obstructive pulmonary disease in general practice: cross sectional case finding study. Bmj.

[33] Price DB, Tinkelman DG, Halbert R, Nordyke RJ, Isonaka S, Nonikov D (2006). Symptom-based questionnaire for identifying COPD in smokers. Respiration.

[34] Price DB, Tinkelman DG, Nordyke RJ, Isonaka S, Halbert R (2006). Scoring system and clinical application of COPD diagnostic questionnaires. Chest.

[35] Kotz D, Nelemans P, Van Schayck C, Wesseling G (2008). External validation of a COPD diagnostic questionnaire. Eur Respir J.

[36] Kawayama T, Minakata Y, Matsunaga K, Yamagata T, Tsuda T, Kinoshita M (2008). Validation of symptom-based COPD questionnaires in Japanese subjects. Respirology.

[37] Stanley AJ, Hasan I, Crockett AJ, Van Schayck OC, Zwar NA (2014). COPD diagnostic questionnaire (CDQ) for selecting at-risk patients for spirometry: a cross-sectional study in Australian general practice. NPJ Prim Care Respir Med.

[38] Frith P, Crockett A, Beilby J, Marshall D, Attewell R, Ratnanesan A (2011). Simplified COPD screening: validation of the PiKo-6® in primary care. Prim Care Respir J.

[39] Haroon S, Jordan R, Takwoingi Y, Adab P (2015). Diagnostic accuracy of screening tests for COPD: a systematic review and meta-analysis. BMJ Open.

[40] Pagano L, McKeough Z, Wootton S, Crone S, Pallavicini D, Chan AS (2020). The feasibility of an innovative GP-physiotherapist partnership to identify and manage chronic obstructive pulmonary disease (INTEGRATED): study protocol. Pilot Feasibility Stud.

[41] Miller MR, Hankinson J, Brusasco V, Burgos F, Casaburi R, Coates A (2005). Standardisation of spirometry. Eur Respir J.

[42] Swets JA (1988). Measuring the accuracy of diagnostic systems. Science.

[43] Fischer JE, Bachmann LM, Jaeschke R (2003). A readers’ guide to the interpretation of diagnostic test properties: clinical example of sepsis. Intensive Care Med.

[44] Sichletidis L, Spyratos D, Papaioannou M, Chloros D, Tsiotsios A, Tsagaraki V (2011). A combination of the IPAG questionnaire and PiKo-6® flow meter is a valuable screening tool for COPD in the primary care setting. Prim Care Respir J.

[45] Akobeng AK (2007). Understanding diagnostic tests 3: receiver operating characteristic curves. Acta Paediatr.

[46] Florkowski CM (2008). Sensitivity, specificity, receiver-operating characteristic (ROC) curves and likelihood ratios: communicating the performance of diagnostic tests. Clin Biochem Rev.

[47] Moore PL (2007). Practice management and chronic obstructive pulmonary disease in primary care. Am J Med.

[48] Haroon S, Jordan RE, Fitzmaurice DA, Adab P (2015). Case finding for COPD in primary care: a qualitative study of the views of health professionals. Int J Chron Obstruct Pulmon Dis.

